# Living Bacterial Hydrogels for Accelerated Infected Wound Healing

**DOI:** 10.1002/advs.202102545

**Published:** 2021-10-31

**Authors:** Zunzhen Ming, Lin Han, Meiyu Bao, Huanhuan Zhu, Sujing Qiang, Shaobo Xue, Weiwei Liu

**Affiliations:** ^1^ Central Laboratory Shanghai Tenth People's Hospital Tongji University 301# Yanchang Middle Road Shanghai 200072 China; ^2^ Department of Laboratory Medicine Shanghai Tenth People's Hospital Tongji University 301# Yanchang Middle Road Shanghai 200072 China; ^3^ Department of Laboratory Medicine Shanghai Skin Disease Hospital Tongji University 200# Wuyi Road Shanghai 200050 China

**Keywords:** antibacterial hydrogels, hydrogel microspheres, living bacteria, microbial competition, wound healing

## Abstract

Damaged skin cannot prevent harmful bacteria from invading tissues, causing infected wounds and even serious tissue damage. Traditional treatments can not only kill pathogenic bacteria, but also suppress the growth of beneficial bacteria, thus destroying the balance of the damaged skin microbial ecosystem. Here, a living bacterial hydrogel scaffold is reported that accelerates infected wound healing through beneficial bacteria secreting antibacterial substances. *Lactobacillus reuteri*, a common probiotic, is encapsulated in hydrogel microspheres by emulsion polymerization and further immobilized in a hydrogel network by covalent cross‐linking of methacrylate‐modified hyaluronic acid. Owing to light‐initiated crosslinking, the hydrogel dressing can be generated in situ at the wound site. This hydrogel scaffold not only protects bacteria from immune system attack, but also prevents bacteria from escaping into the local environment, thus avoiding potential threats. Both in vitro and in vivo experiments show that it has excellent ability against harmful bacteria and anti‐inflammatory capabilities, promoting infected wound closure and new tissue regeneration. This work may open up new avenues for the application of living bacteria in the clinical management of infected wounds.

## Graphical abstract

A convenient and novel living bacteria hydrogel platform is presented, which not only provides protection for bacteria from the immune system, but also prevents bacteria from escaping into the local environment, avoiding the potential threats. In vitro and in vivo experiments, the platform exhibits excellent ability against harmful bacteria and anti‐inflammatory capabilities, promoting infected wound closure and new tissue regeneration.

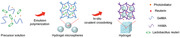

## 1 Introduction

The skin plays an extremely important role in preventing water loss and blocking the invasion of harmful substances and pathogenic microorganisms as a significant interface between the body and its surroundings, where a variety of microbial communities co‐exist, including bacteria, fungi, and viruses.^[^ ^1^, ^2^, ^3^ ^]^ In particular, diverse microbial communities have significant impacts on local and systemic immune responses through interactions with host epithelial and immune cells.^[^ ^3^, ^4^, ^5^, ^6^ ^]^ However, once the skin is damaged, it provides an opportunity for harmful bacteria to invade viable tissues, causing wound infections and even serious tissue damage.^[^ ^7^ ^]^ To address the dilemma of bacterial infection, extensive efforts have been made, as reflected by the constant updates of antimicrobial agents and antimicrobial materials, including new antibiotics, antibacterial nanoparticles, cationic polymer compounds, and antimicrobial peptides.^[^ ^8^, ^9^, ^10^, ^11^, ^12^, ^13^, ^14^, ^15^, ^16^, ^17^, ^18^ ^]^ Although various strategies have achieved encouraging performance in terms of inhibiting bacterial growth and accelerating skin wound healing, it is worth noting (but easily ignored) that these antimicrobial substances kill pathogenic bacteria while suppressing the growth of beneficial bacteria, thereby destroying dermal microbial ecosystem balance.^[^ ^19^ ^]^ Therefore, it would be highly desirable and significant to develop an alternative approach for suppressing the reproduction of deleterious bacteria without inhibiting the growth of beneficial bacteria, but this has proven to be difficult.

Living bacterial therapy, which has been used to treat a variety of inflammatory and immunologic pathologic diseases by bacterial interference and immunomodulation, has received much attention over the past few years.^[^ ^20^, ^21^ ^]^ In nature, microorganisms compete for limited nutrition or space to survive.^[^ ^22^ ^]^ In particular, certain beneficial bacteria can create unique local microenvironments by secreting large amounts of metabolites and antimicrobial agents, which are suitable for their own survival but which inhibit the growth of competing microorganisms.^[^ ^23^, ^24^ ^]^ Thus, beneficial bacteria that secrete biologically active substances with antibacterial, anticancer, or immunosuppressive capabilities have been extensively explored for diagnosis and treatment.^[^ ^25^, ^26^, ^27^ ^]^ For example, *Lactobacilli* have been successfully utilized to treat urinary tract infections by restoring the natural vaginal flora.^[^ ^28^ ^]^ Furthermore, *Bacillus subtilis* has been employed as a producer of antifungal agents for treating fungal infections.^[^ ^29^, ^30^ ^]^ However, the application of bacteria has been largely limited due to the intrinsic nature of bacteria, including rapid proliferation and colonization, which may result in uncontrollable invasion.^[^ ^31^, ^32^ ^]^ In addition, exposed bacteria often cause attacks by the immune system and cannot achieve desired therapeutic effects.^[^ ^33^ ^]^ Therefore, it is challenging to design a scaffold that can protect bacteria from attack by the immune system, using the bioactive substances secreted by bacteria, and thereby prevent bacteria from escaping into the local environment and causing serious infections.

**Scheme 1:**
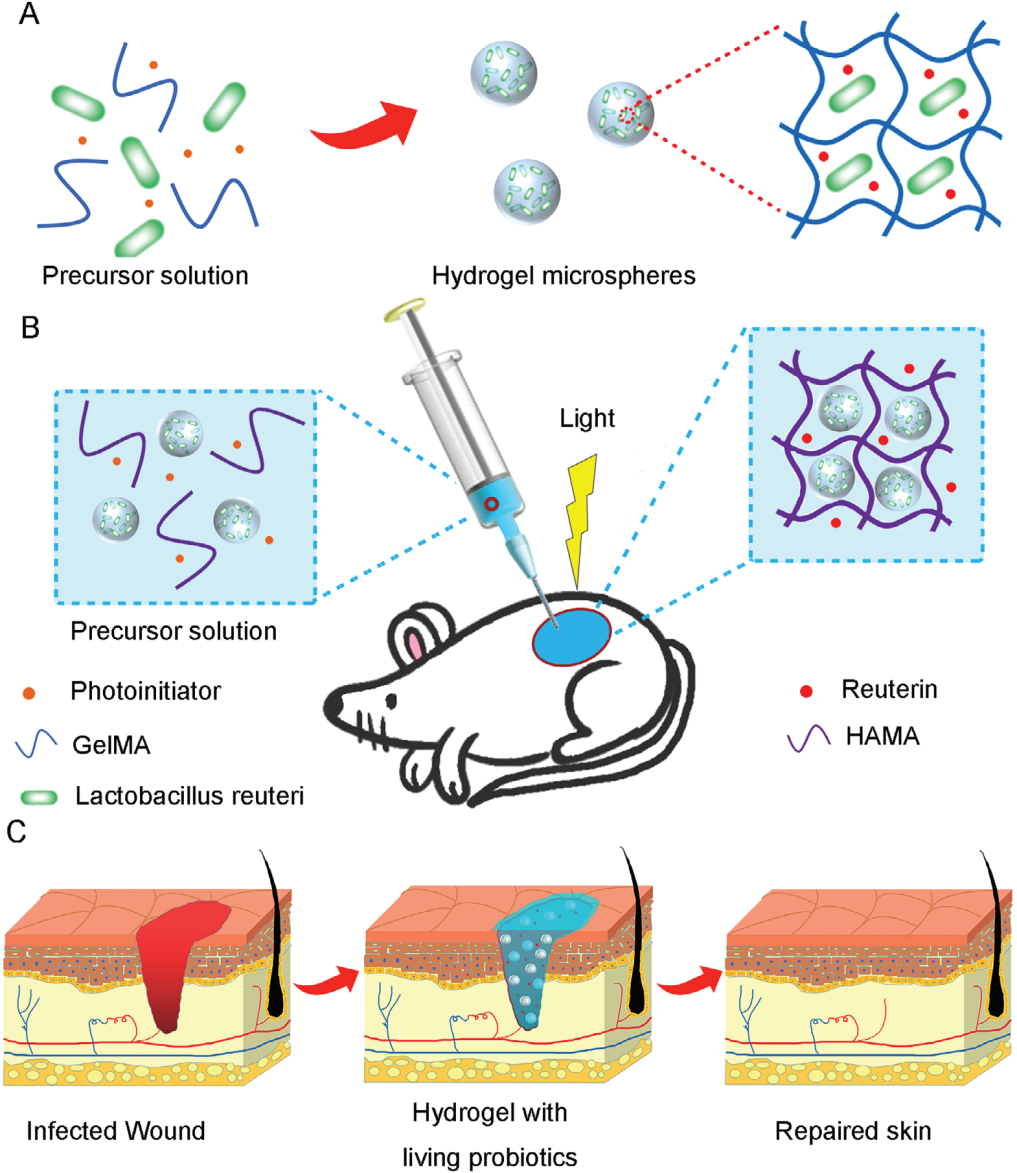
Schematic illustrations of the preparation of living probiotics hydrogels and the process of accelerating the wound healing. A) The process of living bacteria encapsulated in microspheres. B) The gelation of microspheres containing living probiotics and HAMA solution in situ by covalent crosslinking under irradiation of light. C) The process of wound healing with living bacteria hydrogel treatment.

Hydrogels, popular biomedical polymers with 3D molecular networks, have been widely applied in the fields of drug delivery, implantation, and tissue engineering.^[^ ^34^ ^]^ Owing to high biocompatibility and a moist healing environment, hydrogels were applied to promote tissue repair and regeneration.^[^ ^35^ ^]^ Hydrogels not only provide physical barriers to prevent bacteria from invading damaged skin, but also absorb excessive wound exudates. Herein, we present a convenient and novel hydrogel scaffold to treat bacterial infections and accelerate wound healing, and which contains living bacteria encapsulated in hydrogel microspheres forming a hydrogel at the wound site. First, *Lactobacillus reuteri*, a heterofermentative lactic acid probiotic, was selected as the living bacterial strain, which has been shown to inhibit the growth of pathogenic bacteria through lower local pH and antibacterial agents produced during metabolism.^[^ ^36^, ^37^, ^38^ ^]^ As is well known, hydrogels can provide good environments for nutrient transfer, cell growth, and sensing, and are widely considered to be ideal materials for encapsulating living cells.^[^ ^39^ ^]^ To prevent the escape of encapsulated bacteria from the hydrogel, as shown in **Scheme** **1**A, *L. reuteri* was wrapped into hydrogel microspheres through emulsion polymerization, which allowed the delivery of certain substances, including nutrients, biomolecules, lactic acid, and antibacterial agents. Then, a hydrogel dressing was formed at the wound site by covalent crosslinking of methacrylate‐modified hyaluronic acid under light irradiation (Scheme 1B,C). It is interesting to note that in vitro experiments demonstrated that the encapsulated bacteria did not escape into the environment, and the hydrogel exhibited good resistance to pathogenic bacteria. Furthermore, lesser inflammation and faster healing were observed at the wound site in mice treated with living bacterial hydrogel, demonstrating the outstanding anti‐infection and accelerated wound healing ability of such materials in vivo. Therefore, we believe that the strategy described here will open a new window for living bacterial therapy as a safer means of therapy, promoting the application of bacteria in the treatment of various diseases.

## 2 Results and Discussion

### 2.1 Preparation and Characterization of Hydrogel Microspheres (GHM‐LR) Encapsulating Living Bacteria

**Figure 1:**
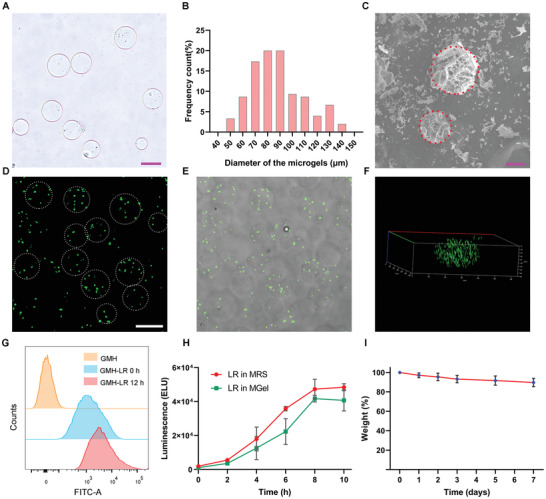
Characterization of hydrogel microspheres (MGel) and bacterial growth in microspheres. A) Representative images of microspheres, scale bar: 100 µm. B) Particle size distribution for microspheres. C) SEM images of microspheres, scale bar: 100 µm. After culturing 24 h in specifically MRS medium, the images of bacteria wrapped in microspheres taken by laser confocal microscope D) Fluorescence field, E) Merge of fluorescence field and bright, and F) 3D mode. G) Representative flow cytometry histogram of microspheres wrapping living bacteria. H) *L. reuteri* viability in microspheres (MGel) compared to the MRS media. I) Stability of microspheres. The weight of freeze‐dried microspheres was a measure to evaluate the degradation of samples in PBS for different times.

Hydrogel microspheres have attracted the attention of researchers in biomedicine and tissue engineering as delivery vehicles for drugs, proteins, and cells because they provide many unique properties compared to bulk hydrogels, including injectability, modularization, and porosity.^[^ ^40^ ^]^ Inspired by hydrogel microspheres encapsulating cells that can protect cells from environmental interference through physical restriction, hydrogel microspheres can prevent the enclosed living bacteria from escaping into the local environment, which provides a safe platform for the growth of bacteria.^[^ ^41^ ^]^ Therefore, in this study, living *L. reuteri* was first successfully encapsulated into hydrogel microspheres by the covalent crosslinking of methacrylated gelatin solution containing living bacteria through emulsion polymerization. Representative images of the hydrogel microparticles are shown in **Figure** **1**A and Figure S1, Supporting Information. The prepared microspheres exhibited highly spherical and clear borders. In addition, as shown in Figure 1B, several representative images were statistically analyzed using ImageJ software to obtain a histogram of the microsphere diameters, indicating the size of most microparticles as ranging from 70 to 90 µm. The morphology of the lyophilized microspheres was obtained by scanning electron microscopy (SEM), which revealed uniform porous structures (Figure 1C). To better visualize the growth state of bacteria in microspheres, images of *L. reuteri* were captured by laser scanning confocal microscopy (LSCM) after staining with a Live/Dead bacterial viability kit. As shown in Figure 1D–F and Figure S2, Supporting Information, a large number of green fluorescent bacteria were observed, demonstrating that the hydrogel microspheres were suitable for bacterial growth because of their excellent biocompatibility. In addition, it can be clearly seen that living bacteria were successfully encapsulated in the hydrogel microspheres. To further explore the proliferation of living bacteria enclosed in microspheres, flow cytometric analysis was performed. As illustrated in Figure 1G and Figure S3, Supporting Information, an obvious shift to a higher mean fluorescence intensity was observed after incubation for 12 h. To quantify the capacity of *L. reuteri* to grow inside the hydrogel microspheres, a viability assay was conducted by measuring luminescence levels. As shown in Figure 1H, compared to the bacteria in DeMan–Rogosa–Sharpe (MRS) medium, *L. reuteri* encapsulated in microspheres showed a similar growth trend. Notably, bacteria embedded in hydrogel microspheres in MRS media exhibited a lower level of luminescence than without bacterial wrapping, which may contribute to the physical limitation of microspheres on the growth of bacteria. Additionally, the hydrogel microspheres also showed good stability over 7 days in PBS, as shown in Figure 1I. Overall, the hydrogel microspheres encapsulating living bacteria were successfully prepared and proved to be suitable for bacterial growth, laying a foundation for subsequent experiments.

### 2.2 Preparation and Characterization of Hydrogel (MHA‐LR) Containing GHM‐LR

**Figure 2:**
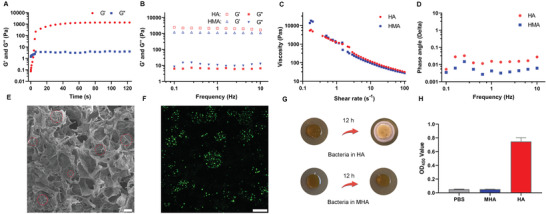
The characterization and ability to prevent bacteria from escaping of hydrogel (MHA‐LR) containing GHM‐LR. A) Rheology analysis indicated the hydrogel (MHA‐LR) formation. B) Frequency‐sweep oscillatory tests. C) Flow curves of the prepared hydrogel. D) Phase angle analysis of HMA and HA hydrogel as a function of frequency. E) SEM images of MHA‐LR, scale bar: 100 µm. F) The images of wrapped bacteria in hydrogels taken by laser confocal microscope, scale bar: 100 µm. G) The culture of *L. reuteri* with (MHA) or without (HA) wrapping by microspheres in hydrogels on an agar plate. H) The OD value at 450 nm of bacteria in MHA or HA after culturing in MRS medium.

Bulk hydrogel containing living bacteria wrapped in microspheres was fabricated using a photocrosslinking reaction between methacrylated hyaluronic acid and microspheres. Furthermore, infrared spectroscopic analysis was further performed to explore the constitution of prepared hydrogels. As shown in Figure S4, Supporting Information, the appeared signals of the prepared hydrogels that attributed to the characteristic absorption of GelMA (1447 cm^−1^) and HAMA (1406 cm^−1^) precursors indicate the successful formation of the corresponding network. Rheological analysis was carried out to explore the gelation time and mechanical properties of the bulk hydrogel (MHA‐LR). As shown in **Figure** **2**A, the hydrogel was rapidly formed in approximately 10 s, and the values of G’ and G’’ also tended to be stable after 40 s, demonstrating the rapid gelation ability of the system and the integrity and stability of the network.^[^ ^42^ ^]^ There was no significant difference between the hydrogel containing microspheres and hydrogels without microspheres (Figure S5, Supporting Information). In addition, hydrogels with or without microspheres exhibited constant values of G’ and G’’ over the frequency range of 0.1 to 10 Hz (Figure 2B), which could be attributed to the covalent crosslinking inherent to the network.^[^ ^43^ ^]^ Furthermore, as shown in Figure 2C, a significant viscosity decrease was observed, displaying a non‐Newtonian or pseudoplastic characteristic with a shear rate ranging from 0.01 to 100 s^−1^. Phase angles of HMA and HA hydrogel were measured as a function of frequency. As shown in Figure 2D, there was no significant difference between the two samples and the phase angle was about 0.01, indicating typical elastic properties of the hydrogels.

SEM images showed that compared with HA hydrogels, porous microspheres were observed in lyophilized hydrogels containing microspheres, which was consistent with the structure of freeze‐dried hydrogel microspheres, as shown in Figure 2E and Figure S6, Supporting Information. To visualize the growth of *L. reuteri* in MHA, images of living bacteria were obtained by LSCM after staining with the Live/Dead Kit. As observed in Figure 2F, living bacteria exhibited a good growth state, indicating that the material was nontoxic to bacteria throughout the preparation process. As mentioned above, hydrogel microspheres not only promoted living bacterial growth as a scaffold, but also prevented bacteria from escaping into the environment and causing potential infection. To verify its ability to prevent bacteria from escaping, the hydrogel (HA) directly mixed with *L. reuteri* and the hydrogel (MHA) containing bacterial encapsulated microspheres were placed on MRS plates and cultured for 12 h. As shown in Figure 2G, a large number of *L. reuteri* colonies were observed around the hydrogel, which could be explained by the proliferation of bacteria exposed to the surface of the material. However, there was no bacterial growth around the hydrogel containing microspheres encapsulating living *L. reuteri*, demonstrating that the microspheres prevented bacteria from escaping into the local environment. In addition, these two hydrogels mixed with living bacteria by different methods were placed in a liquid MRS medium for incubation for 12 h, and the number of bacteria in the culture medium was evaluated by reading the optical density at 450 nm after incubation with the Microbial Viability Assay Kit‐WST. As seen in Figure 2H, the OD value for the HA‐soaked solution was up to 0.8, suggesting the proliferation of numerous bacteria. However, the OD value of the MHA‐soaked solution was almost the same as that in PBS. In addition, we replaced *L. reuteri* with *Escherichia coli* to perform the above experiments, and the same phenomena and results were observed, as shown in Figure S7, Supporting Information. These results confirmed that hydrogels containing living bacteria encapsulated in microspheres exhibited excellent biosafety to avoid potential bacterial infection.

### 2.3 Antibacterial Activity and Biocompatibility Evaluation In Vitro

**Figure 3:**
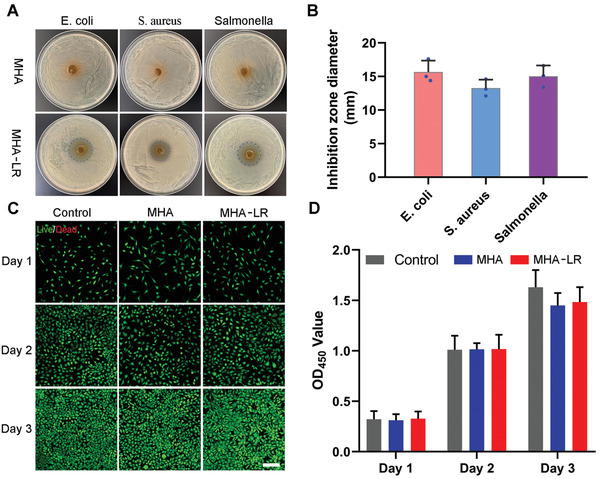
The antibacterial ability and biocompatibility of hydrogels containing *L. reuteri* encapsulated microspheres in vitro. A) Antibacterial sensitivity of hydrogels with (MHA + LR) and without (MHA) *L. reuteri* against *E. coli*, *S. aureus*, and Salmonella with agar diffusion test. B) Inhibition zone diameters for MHA + LR. C) Representative pictures of control, MHA, and MHA + LR taken by a confocal laser microscope after staining with Live/Dead Kit for 1, 2, and 3 days. D) The OD value of different groups at 450 nm after incubating with CCK‐8 Kit.

*L. reuteri*, a probiotic commonly found in humans and animals, can protect against the adverse effects of certain microorganisms and modulate immune responses.^[^ ^34^, ^35^, ^36^ ^]^ According to previous reports, *L. reuteri* can produce lactic acid to reduce the pH of the local environment, which inhibits the growth of harmful bacteria such as *Staphylococcus aureus*.^[^ ^34^ ^]^ In addition, *L. reuteri* can also secrete a potent antimicrobial agent called reuterin against gram‐positive and gram‐negative bacteria. To confirm the presence of reuterin in hydrogels containing *L. reuteri*, the hydrogels were soaked in an MRS medium for 12 h and the co‐cultured solution was analyzed using electron ionization (EI) mass spectrometer. As shown in Figure S8, Supporting Information, the molecular ion peak of reuterin was observed at m/z 74, indicating the presence of an antimicrobial agent. Therefore, we expected hydrogels containing *L. reuteri* encapsulated in microspheres to exhibit good antibacterial activity against harmful bacteria, including *E. coli, S. aureus*, and *Salmonella spp*. In vitro antimicrobial tests were performed to evaluate the antibacterial activity of the materials. Compared with hydrogel fabricated without *L. reuteri*, obvious inhibition zones were observed on the agar plate on which the MHA‐LR was placed for 12 h, as illustrated in **Figure** **3**A. The zone diameter for the MHA‐LR group was 15.1 ± 1.5, 13.2 ± 1.8, and 15.1 ± 1.6 mm against *E. coli, S. aureus*, and *Salmonella*, respectively (Figure 3B). Moreover, the antimicrobial ability of living bacterial hydrogel was evaluated in vitro against *S. aureus* and *E. coli*. As shown in Figure S9, Supporting Information, compared with untreated *S. aureus* and *E. coli*, the vast majority of bacteria were killed after culturing with *L. reuteri* encapsulated hydrogels culture medium, demonstrating the excellent antibacterial ability of materials. It is consistent with the results of the zone of inhibition assay. To further explore the biofilm inhibition properties of living bacterial hydrogel, *S. aureus* was selected as a model bacteria to perform a LIVE/DEAD staining assay. As shown in Figure S10, Supporting Information, the *S. aureus* without being treated with the secretion of living *L. Reuteri* encapsulated in hydrogel exhibited strong green fluorescence, indicating good bacterial activity. On contrary, the treated *S. aureus* showed significant red fluorescence, suggesting that most of the pathogenic bacteria were killed. Given that propidium iodide penetrates only bacteria with damaged membranes, the strong red fluorescence also demonstrated that the membrane of *S. aureus* was damaged. These results indicated that MHA‐LR exhibited excellent capability against various harmful bacteria, which could be ascribed to the lactic acid and reuterin secreted by *L. reuteri* within hydrogels.

To evaluate the cytocompatibility of MHA and MHA‐LR, mouse fibroblast L929 cells were chosen as a model to perform live/dead assays. As shown in Figure 3C, a large number of living cells was observed in the three groups during the whole period, and there were no significant differences among them after incubation for 24, 48, and 72 h. This showed that MHA and MHA‐LR gels did not affect cell growth and proliferation, demonstrating their excellent biocompatibility. Notably, living *L. Reuteri* wrapped in microspheres in the hydrogel had almost no negative effect on cell growth, which could be explained by the microspheres preventing the bacteria from escaping into the environment. To further assess the cytocompatibility of the materials, quantification data were obtained using the Cell Counting Kit 8 kit (CCK8). As illustrated in Figure 3D, there were no obvious differences in the OD values among the three groups, indicating no significant cytotoxic effects of MHA and MHA‐LR. These results suggested that hydrogels containing living bacteria within microspheres exhibited good biocompatibility without causing side effects in terms of cell growth and proliferation, and could be expected to be used in vivo for further biological applications.

### 2.4 Antibacterial Activity and Biocompatibility Evaluation In Vivo

**Figure 4:**
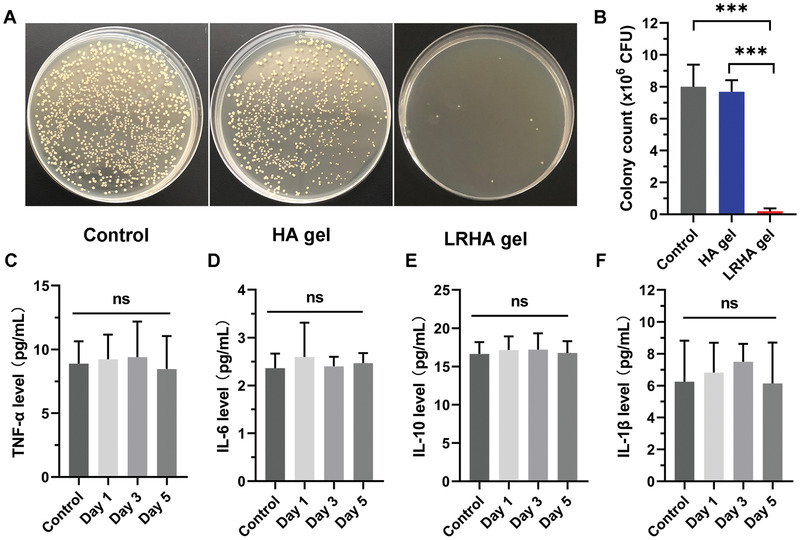
Antibacterial activity and biocompatibility evaluation in vivo. A) Representative photos of S. aureus colonies on agar broth plates separated from infected wounds with different treatments. B) The colony count of S. aureus from different groups. C–F) The concentrations of TNF‐*α* (C), IL‐6 (D), IL‐10 (E), and IL‐1*β* (F) in control and implanted hydrogels containing living *L. reuteri* mice.*p < 0.05, **p < 0.01, and ***p < 0.001.

To evaluate the antibacterial ability of living bacterial hydrogel in vivo, the bacteria were separated from the infected wound after two days of treatment and was spread on agar broth plates to culture for 24 h. As shown in **Figure** **4**A,B, a lot of bacteria were observed in the control and HA treatment groups, indicating high bacterial growth in infected tissue. However, the infected skin showed a small amount of bacterial growth after treating with living *L. reuteri* hydrogel, suggesting the dress's obvious antibacterial activity in vivo.

Moreover, the biocompatibility of living bacterial hydrogel was evaluated by analyzing inflammatory cytokine of mice implanted with materials containing living *L. reuteri*. On days 1, 3, and 5, the inflammatory cytokine including TNF‐*α*, IL‐6, IL‐10, and IL‐1*β* was measured by using corresponding ELISA Kits. As shown in Figure 4C–F, there was only a slight difference in all inflammatory factors between the control group and the implanted living bacterial hydrogel group, demonstrating good biocompatibility of designed materials in vivo.

### 2.5 In Vivo Evaluation of Wound Healing

**Figure 5:**
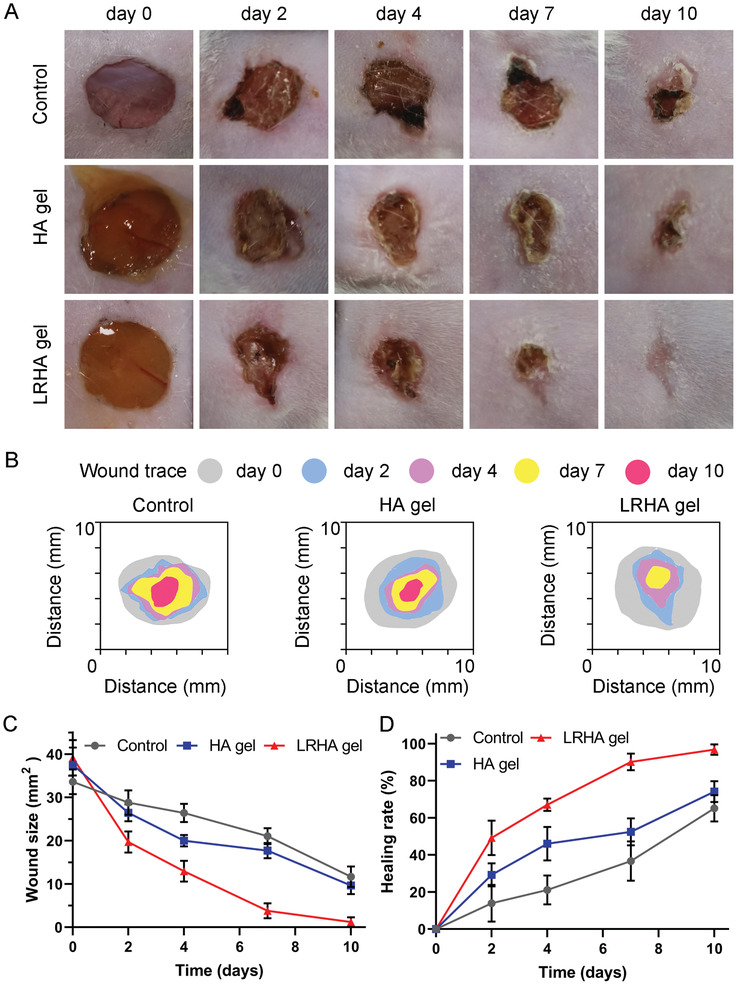
In vivo infected wound healing studies. A) Representative pictures of wound tissues of different groups on days 0, 2, 4, 7, and 10. B) Traces of wound‐bed closure during 10 days for three groups. C) Wound size over time in mice treated with different methods. D) Wound healing rate for different groups during treatment.

A full‐thickness cutaneous wound model with *S. aureus* infection was established to evaluate the efficiency of hydrogels in promoting wound healing. Mice were divided into three groups for in vivo testing: no treatment (control group), HA hydrogel treatment (HA group), and HA hydrogels containing microsphere encapsulating living *L. reuteri* treatment (LRHA group). As shown in **Figure** **5**A, the process of wound healing was monitored using images captured with a digital camera. In the control group, yellow pus was observed at the wound site on days 2 and 4, and the injury was bright red, indicating that the damaged site was undergoing inflammation. However, there was almost no yellow pus in the LRHA group during the entire treatment period compared with the HA and control groups. In addition, the wounds of mice treated with LRHA exhibited accelerated wound closure on day 2, indicating that infections caused by *S. aureus* were controlled (Figure 5B). By day 4, the wound size of mice treated with LRHA dropped significantly from 38.5 ± 2.3 to 14.8 ± 2.6 mm^2^, in which the wound healing rate reached 64% (Figure 5C,D). However, the wound sizes of control and HA group animals were still 28.3 ± 2.1 and 21.5 ± 1.8 mm^2^, respectively, with wound healing rates of 20% and 42%, respectively. After 10 days of treatment, wounds treated with LRHA were completely closed and covered with new epidermal tissue. However, 38% and 24% of the wounds remained unhealed after no treatment and treatment with HA, respectively. The wound closure time was extended to 15 and 13 days in the control and HA groups, respectively, as shown in Figure S11, Supporting Information. These results suggested that LRHA plays an important role in promoting wound healing, which is attributed to the excellent antibacterial ability of living *L. reuteri* secreting lactic acid and antimicrobial agents. It is worth noting that the HA‐treated wounds showed a faster healing rate than the control group, which could be explained by the hydrogel having a certain promoting effect on wound healing and providing a physical barrier to prevent bacteria from invading adjacent tissues.

**Figure 6:**
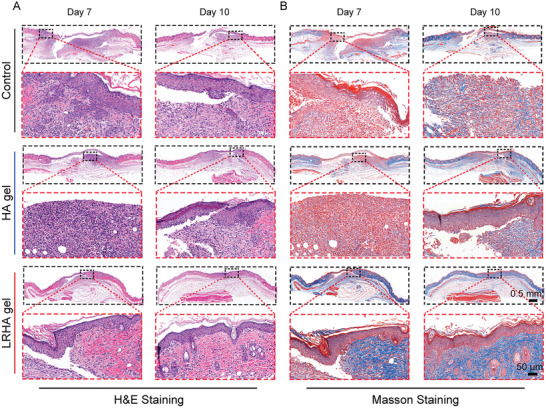
Histopathologic profiles of the wound for different groups. A) Tissue slices stained with Hemotoxylin and eosin (H&E) from different groups on days 7 and 10. B) Masson's trichrome stained wound tissue slices for different groups on days 7 and 10.

To further evaluate the process of wound healing, a series of pathological examinations were performed, including hematoxylin and eosin (H&E) staining, Masson's trichrome staining, and Sirius red staining. As shown in **Figure** **6**A, tissues in the wounds of the control, HA, and LRHA groups were stained with H&E on days 7 and 10. In the control and HA groups, large numbers of inflammatory cells were observed on day 7, indicating that serious inflammation was still not controlled due to the absence of antibacterial activity of the treatments. In addition, there was almost no new epidermal tissue in either group. However, only a small number of neutrophils appeared by day 7 in the wound tissues of mice treated with LRHA, which was ascribed to the excellent capability against *S. aureus* of living *L. reuteri* wrapped in LRHA. Notably, new blood vessels and regenerated epidermis were observed, demonstrating that wound healing was accelerated by LRHA treatment. After 10 days of treatment, the defective skin was healed completely in the LRHA group, and was almost the same as normal skin (Figure S12, Supporting Information), indicating the regeneration of dermal tissue. However, there were many inflammatory cells and an unclosed wound in the control and HA groups. Moreover, Masson's trichrome staining and Sirius red staining showed that the renewed collagen gradually increased the healing time for the three groups (Figure 6B, Figures S13 and S14, Supporting Information). However, more regenerated collagen was deposited in the wounds treated with LRHA than in the defects treated in the other two groups, further confirming the superior wound healing effects of LRHA.

**Figure 7:**
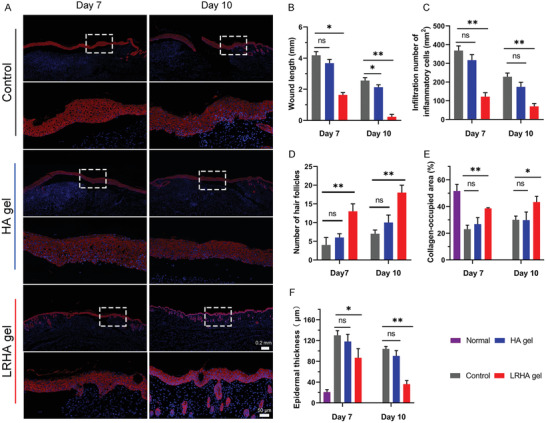
A) Representative images of immunohistochemistry staining with cytokeratin 14 for different groups on days 7 and 10. Quantification of histopathologic analysis of different groups on days 7 and 10 B) wound size, C) infiltration numbers of inflammatory cells, D) number of hair follicles, E) collagen‐occupied area, and F) epidermal thickness. *p < 0.05, **p < 0.01, and ***p < 0.001.

Furthermore, the renascent epithelial thickness was investigated by staining the wound tissue for cytokeratin 14 (CK14) after 7 and 10 days of treatment using different methods. As shown in **Figure** **7**A, there was no significant difference in epithelial thickness between the control and HA groups. During the whole wound healing process, the renascent epithelial thickness became thinner and thinner for all three groups with an increase in repair time. However, the epithelial thickness of the LRHA‐treated groups was as thin as that of normal tissue (Figure S15, Supporting Information), while there was a relatively full thickness in the control and HA groups, suggesting that LRHA promoted the wound healing process. Further quantification was performed to explore the effects of LRHA on accelerating wound healing (Figure 7B–F). Compared with the control and HA groups, the wound tissues of mice in the LRHA group exhibited shorter wound lengths, fewer inflammatory cells, a larger number of hair follicles, a higher ratio of collagen‐occupied regions, and a thinner epithelial layer after 7 and 10 days of treatment. In addition, to explore the toxicity of materials in vivo, the major organs of the three groups were harvested and analyzed by H&E staining, involving the heart, liver, spleen, lungs, and kidneys. As shown in Figure S16, Supporting Information, histological analysis showed that these major organs maintained the integrity of the tissue without abnormal defects or damage, which was not significantly different from normal tissues, demonstrating the excellent biocompatibility of LRHA. These results demonstrated that LRHA has excellent antibacterial ability and outstanding performance in accelerating wound healing, which provides a new and safe strategy based on living bacteria for potential applications in wound repair in the clinic.

## 3 Conclusions

In summary, we successfully encapsulated live *L. reuteri* into microspheres by emulsion polymerization involving methacrylate gelatin and synthesized hydrogel dressings in situ by covalent crosslinking of methacrylate hyaluronic acid to accelerate wound healing. Encapsulated living bacteria within microspheres can grow and proliferate normally in the hydrogel, secreting lactic acid and antibacterial agents into the local environment. This hydrogel dressing not only protects bacteria from the immune system, but also prevents bacteria from escaping into the local environment, avoiding potential threats. In in vitro experiments, LRHA exhibited excellent antibacterial effects against different bacteria and with outstanding biocompatibility. Subsequently, in vivo experiments further revealed that LRHA can mitigate inflammatory cell infiltration, enhance collagen deposition, and accelerate wound healing. We anticipate that this work will open a window for the application of living bacteria in the treatment of infected wounds and tissue engineering.

## 4 Experimental Section

### The Preparation of HA‐MA

The methacrylated hyaluronic acid was prepared according to previous literature reports. First, 2 g hyaluronic acid was dissolved in 100 mL deionized water and the pH of the solution was adjusted to 8. An excess of methacrylic anhydride was added to the solution and the mixed solution was stirred for 12 h at room temperature. Then, the product of HA‐MA was obtained by ethanol precipitation and washed with ethanol three times to remove the remaining methacrylic acid and methacrylic anhydride. ^1^H‐NMR spectra of HA‐MA were recorded on a Bruker Avance 400 MHz spectrometer (Figure S17, Supporting Information).

### The Preparation of *Lactobacillus Reuteri*‐Loaded Gelatin Hydrogel Microparticles (LRGHM)

*L. reuteri* was successfully wrapped into the hydrogel microspheres through the emulsion polymerization of methacrylate modified gelatin. Specifically, 60 mg methacrylated gelatin were dissolved in 1 mL PBS containing 10^8^ *L. reuteri* and 3 mg LAP to prepare a hydrogel precursor solution. Under the irradiation of 365 nm wavelength light (10 mW cm^−2^), the mixture solution was added to 3 mL mineral oil containing 2% Span 80 in a glass flask with constant stirring. Light exposure was limited to 1 min, since hydrogel microspheres settle out of solution rapidly. *L. reuteri*‐loaded hydrogel particles were then separated from the mixture system by centrifuging at 300 g for 2 min. Finally, gelatin microparticles containing *L. reuteri* were washed three times by 1× PBS and then resuspended to PBS for the follow‐up study.

### The Preparation of Hydrogel

HA hydrogel containing *L. reuteri*‐loaded gelatin hydrogel particles (LRHA gel) were fabricated by the following method. In particular, 15 mg HA‐MA and 3 mg LAP were added to 1 mL PBS containing LRGHM to prepare a hydrogel precursor solution. Then, the precursor was poured into Teflon disc molds and was irradiated with 365 nm wavelength LED for 5 s to obtain the LRHA gel. HA hydrogel (HA gel) without *L. reuteri* were prepared with the same method.

### Particle Size Statistics of Gelatin Microspheres

LRGHM were diluted into the 1× PBS in a 50 mm petri dish with a suitable amount. 30 images were taken randomly by a Leica fluorescence microscopy under white light channel. The size distribution of gelatin microspheres was quantified and analyzed by ImageJ Software.

### The Stability Test of Gelatin Microspheres

The obtained gelatin microspheres were immersed in PBS solution at room temperature. At a certain time, the weight of microspheres was recorded after centrifugation at 300 g for 2 min and resuspended into a PBS solution. The stability of gelatin microspheres was evaluated by the following formula:

(1) $$\text{Remaining}\;\text{percentage}\left(\%\right)=\frac{w}{w_{0}}\;\times 100\%$$

Where, *w* means the weight of gelatin microspheres and *w*_0_ means the initial weight of gelatin microspheres.

### Bacterial Culture

*L. reuteri* were cultured in MRS medium supplemented with 0.05% L‐cysteine and filter‐sterilized 20 mm glycerol at 37 ℃. *E. coli*, *S. aureus*, and Salmonella were cultured in LB broth in a shaker at 37 ℃.

### Morphological Characterization of Hydrogel Microparticles and Hydrogel

The obtained gelatin microspheres, HA hydrogel, and LRHA hydrogel were freeze‐dried in a lyophilizer (scientz‐12ND/C, China) for 2 days. The morphologies of the prepared samples were observed by a field emission scanning electron microscope (FE‐SEM, Sirion 200) at an accelerating voltage of 3 kV.

### Fluorescence Images of Bacteria

The gelatin microspheres encapsulating *L. reuteri* were incubated for 30 min at room temperature in PBS containing the live/dead bacterial staining reagent. Then, the staining gelatin microspheres were washed in PBS to remove the excess staining reagent and imaged by LSCM (Zeiss LSM 900).

### Bacterial Growth Activity

The gelatin microspheres containing *L. reuteri* 10^8^ CFU mL^−1^ were cultured in MRS broth supplemented with 0.05% L‐cysteine at 37 ℃. As a control, the *L. reuteri* (10^8^ CFU mL^−1^) were cultured with the same conditions. At a certain time, eighty microliter samples were incubated with a BacTiter‐Glo Cell Viability Assay kit and placed in opaque all‐white 96‐well plates. Luminance was measured by a multi‐mode microplate reader (Molecular Devices SpectraMax iD5, USA).

### Flow Cytometry

The growth of *L. reuteri* in gelatin hydrogel microspheres was evaluated by examining the fluorescence intensity of microspheres. Specially, the gelatin hydrogel microspheres containing 10^8^ CFU mL^−1^ cells were cultured in MRS broth supplemented with 0.05% L‐cysteine at 37 ℃ for 12 h. Then, the gelatin microspheres were stained with the Live Bacterial Staining Kit after centrifugation and redispersion. Single hydrogel microparticles suspensions were prepared from these samples which were filtered through a 100‐micron sieve and examined by flow cytometry. Flow data were analyzed by FlowJo software.

### Rheology Test

The rheological properties of hydrogels (LRHA gel) were explored by a stress‐controlled rheometer (HAAKE MARS III) with a 20 mm parallel plate and OmniCure Series 2000. Time‐sweep oscillatory tests were performed at a 10% strain and 1 Hz frequency. The gap between the upper plate and the sample dish was set to 0.5 mm and the light irradiation intensity was set to 30 mWcm^−2^ for 300 s. Frequency‐sweep oscillatory tests were recorded in a constant stress (10 Pa) mode over the frequency range of 0.1–50 Hz to maintain the measurements within the linear range. All the experiments were performed in triplicate.

### Biosafety of *Lactobacillus Reuteri* Wrapped Gelatin Hydrogel Microspheres

The prepared hydrogel containing *L. reuteri* wrapped gelatin hydrogel (LRHA gel) microspheres were placed on the MRS agar overnight in the absence of oxygen to observe the growth of microorganisms on agar medium. In addition, prepared LRHA gels were soaked in MRS broth supplemented with 0.05% L‐cysteine at 37 ℃ overnight. Then, the cultured MRS broth was poured into a centrifuge tube and centrifuged at 7000 g for 5 min. After discarding the supernatant, the precipitation was incubated with Microbial Viability Assay Kit‐WST for 2 h to read the optical density at 450 nm by a microplate reader. The hydrogel directly mixed with *L. reuteri* was used as a control.

### In Vitro Antibacterial Test

To explore the antibacterial activity of LRHA gels, an inhibition zone assay was carried out. Specifically, *E. coli*, *S. aureus*, and Salmonella were selected as a model of inhibited bacteria. First, the inhibited bacteria were evenly spread on the surface of the agar medium for inoculation. Then, the prepared LRHA gels with a diameter of 5 mm were placed on the surface of the culture medium inoculated with bacteria for 12 h at 37 ℃. The inhibition zone was measured with a digital vernier caliper to measure the diameter of the entire inhibition zone. To further explore the antimicrobial ability of living bacteria hydrogel, the spread plate experiment was carried out. Specifically, the prepared hydrogel containing *L. reuteri* was soaked in MRS broth supplemented with 0.05% L‐cysteine at 37 ℃ overnight. Then, the stock suspensions (1.0 × 10^6^ CFU mL^−1^) of *E. coli* and *S. aureus* were incubated with living bacterial hydrogel culture medium for 2 h at 37 ℃. The solutions were evenly spread onto LB agar plates after diluting 500 times. Finally, the number of CFUs on the agar plates was counted after culture for 24 h at 37 ℃. In addition, the treated or untreated *S. aureus* was stained with a LIVE/DEAD *BacLight* Bacterial Viability Kits (SYTO 9 green‐fluorescent nucleic acid stain and the red‐fluorescent nucleic acid stain, propidium iodide) for taking pictures by laser confocal microscope.

### Inflammatory Cytokine Analysis In Vivo

First, the prepared hydrogel containing *L. reuteri* was implanted subcutaneously in mice. Blood of mice in different groups was collected into 1 mL centrifuge tubes without additives on 1 d, 3 d, and 5 d. The blood sample was clotted for 30 min at room temperature and was then centrifuged with 2000 xg for 10 min at 4 ℃. The serum was obtained and saved at −80 °C until analysis. All inflammatory were analyzed by ELISA Kits.

### Cell Culture and Cytotoxicity Assay

Mouse fibroblasts L929 cells were chosen as the models to evaluate the biocompatibility of LRHA gels. L929 cells were cultured in Dulbecco's Modified Eagle Medium (DMEM; Gibco) supplemented with 10% (v/v) inactivated FBS (Gibco) and 1% (v/v) antibiotic/anti‐mycotic solution (AB/AM; Sigma). For cytotoxicity assay, L929 cells were added to 96 well plates at a density of 2000 mL^−1^ and incubated with the leach liquor of hydrogels. After certain periods of incubation, CCK‐8 reagent was added to 96 well plates for culturing at 37 ℃ incubator with 5% CO_2_ for 2 h. The cell viability and proliferation were quantified by a multi‐mode microplate reader at 450 nm. In order to better observe the growth of cells, L929 cells were incubated with Live/Dead reagent (Calcein‐AM/PI) for 15 min at 37 ℃. The fluorescence images were taken by LSCM (Zeiss LSM 900).

### In Vivo Wound Healing Assay

For wound healing experiment, 18 male Balb/c mice were selected to evaluate the wound‐healing efficiency of LRHA gels. All mice were randomly divided into 3 groups: the control group, HA group, and LRHA group. Namely, the control group did not undergo any treatment except for wrapping gauze; HA group was treated with HA hydrogel containing the gelatin microspheres without *L. reuteri*; LRHA group was covered with LRHA gels. All procedures involved in animal experiments were approved by the Animal Ethics Committee of Shanghai Tenth People's Hospital.

After anaesthetization with 1% pentobarbital (50 mg kg^−1^) by intraperitoneal injection, the fur of the dorsal skin in all mice was shaved and the remaining hair was completely removed by applying depilatory cream for 10 min. Two circular full‐thickness wounds about 7 mm in diameter were created on the back of each rat. Subsequently, 50 µL of S. aureus suspension (1.0 × 10^6^ CFU mL^−1^) were inoculated into the created wound. The hydrogel microspheres wrapping *L. reuteri* have already been incubated in MRS broth supplemented with 0.05% L‐cysteine and 200 mm glycerol for 24 h before forming a hydrogel dressing at the wound. Hydrogel precursor solution (HA and LRHA) were prepared and added to a one‐time use syringe in advance. Then, precursor solution was injected into the wound and spread evenly. The hydrogel was formed at the wound site by irradiation of 365 nm for 5 s. Finally, the wound covered by hydrogels was wrapped with sterile gauze. Furthermore, 3 µL of the exudate was collected from the infected tissue on day 2 and then diluted 200 times with PBS. Then, 20 µL of each solution was evenly spread onto LB agar plates and cultured at 37 °C for 8 h. All mice were placed in individual ventilated cages to prevent mutual interference. The dressing was changed every 3 d and pictures of the wounds were taken on days 0, 2, 4, 7, 10, and 14 by a digital camera. The obtained images were analyzed by the Image J software. The rate of wound healing in different groups was calculated according to the following formula:

(2) $$\text{Healing}\;\text{rate}\left(\%\right)=\frac{s_{0}-s_{t}}{s_{0}}\;\times 100\%$$

Where, *s*_0_ means the initial area of wound and *s*_t_ means remaining wound area at each time point.

### Histopathological Study

Wound histology specimens were collected on days 7 and 14. All the harvested samples were fixed in 4% paraformaldehyde solution for 12 h and embedded in paraffin to prepare 5 µm thickness tissue sections. Representative specimens were stained with haematoxylin and eosin (H&E), Masson trichrome, and Sirius red to observe the histological images by microscopy. In addition, immunofluorescence staining (CK14) was also performed to evaluate the collagen deposition and angiogenesis. To evaluate the acute toxicity of the material in mice, the major organs were collected and stained with H&E for histological analysis, which included the heart, spleen, liver, kidneys, and lungs.

### Statistical Analyses

All data were presented as means ± standard deviation (SD) based on experiments performed in triplicate or more. Statistical analysis was performed with Graphpad software. Unpaired two‐tailed Student's t‐test was performed for two‐group comparisons. Statistical significance was analyzed by one‐way ANOVA for more than two groups. Statistically significant differences were represented with *p < 0.05, **p < 0.01, and ***p < 0.001.

## Conflict of Interest

The authors declare no conflict of interest.

## Supporting information

Supporting Information

## Data Availability

The data that supports the findings of this study are available in the article and in the Supporting Information.
