# Supplementary material for: Living Bacterial Hydrogels for Accelerated Infected Wound Healing
Source: Adv Sci (Weinh). 2021 Oct 31;8(24):2102545. doi: 10.1002/advs.202102545 (PMC8693052; doi:10.1002/advs.202102545)
Supplement: Supplementary file 1 — Supporting Information [file ADVS-8-2102545-s001.pdf]

© 2021 Wiley-VCH GmbH

Supporting Information

for *Adv. Sci.,* DOI: 10.1002/advs.202102545

Living Bacteria Hydrogels for Accelerating Infected Wound Healing

Zunzhen Ming, Lin Han, Meiyu Bao, Huanhuan Zhu, Sujing Qiang, Shaobo Xue, Weiwei Liu**

Supporting Information

Living Bacteria Hydrogels for Accelerating Infected Wound Healing

Zunzhen Ming, Lin Han, Meiyu Bao, Huanhuan Zhu, Sujing Qiang, Shaobo Xue, Weiwei Liu**

Materials: Hyaluronic acid (HA) with a molecular weight (MW) of 100,000 was supplied from Sigma-Aldrich. Methacrylic anhydride (MA) and methacrylate gelatin (GelMA) were purchased Shanghai Aladdin Biochemical Technology Co. Ltd. Lithium Phenyl(2,4,6-trimethylbenzoyl)phosphinate (LAP), Span 80, mineral oil were obtained from Sigma-Aldrich. Dulbecco's phosphate buffered saline (PBS) and other chemical reagent (Adamas-beta®) were purchased from Titan. Microbial Viability Assay Kit-WST, Calcein-AM/PI Double Staining Kit and Cell Counting Kit-8 (CCK-8) were purchased from Dojindo Chemical Technology (Shanghai) Co. Ltd. A BacTiter-Glo Microbial Viability Assay kit was supplied from Promega. Dulbecco's Modified Eagle Medium (DMEM) and Fetal Bovine Serum (FBS) were purchased from Thermo Fisher Technology (China) Co., Ltd. The ELISA Kits were purchased from MULTISCIENCES（LIANKE) BIOTECH,CO.,LTD. Luria Bertani agar/broth (LB) and DeMan-Rogosa-Sharpe medium (MRS) were purchased from Beijing Solarbio Science & Technology Co., Ltd. Lactobacillus reuteri, Escherichia coli, Staphylococcus aureus and Salmonella were obtain from the American Type Culture Collection (ATCC). Mouse fibroblasts L929 cells (L929) were obtained from

American Type Culture Collections (ATCC). All Balb/c mice were supplied from Beijing Vital River Laboratory Animal Technology Co. Ltd.

Results

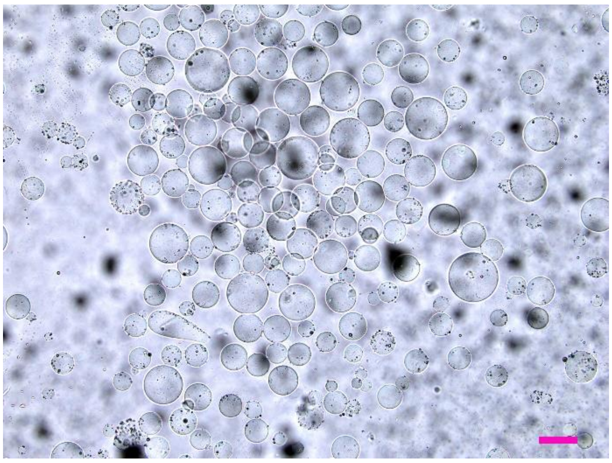

Figure S1. The representative images of the hydrogel microspheres. Scare bar: 100 μm.

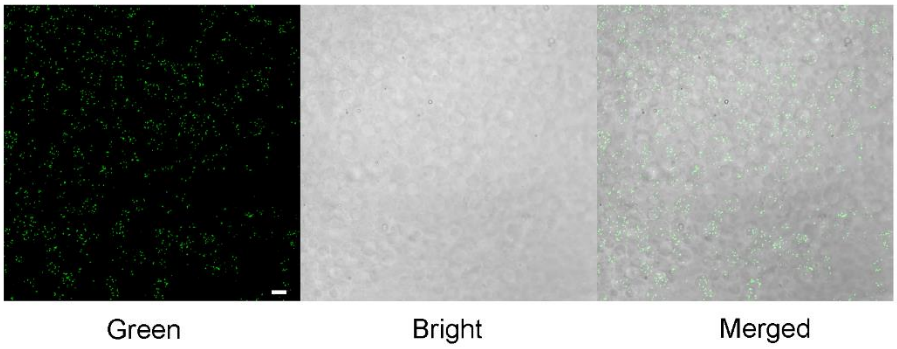

Figure S2. The images of bacteria wrapped in microspheres taken by laser confocal microscope.

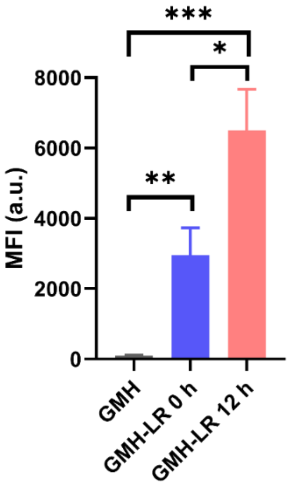

Figure S3. MFI quantifications of microspheres wrapping living bacteria by flow cytometry.

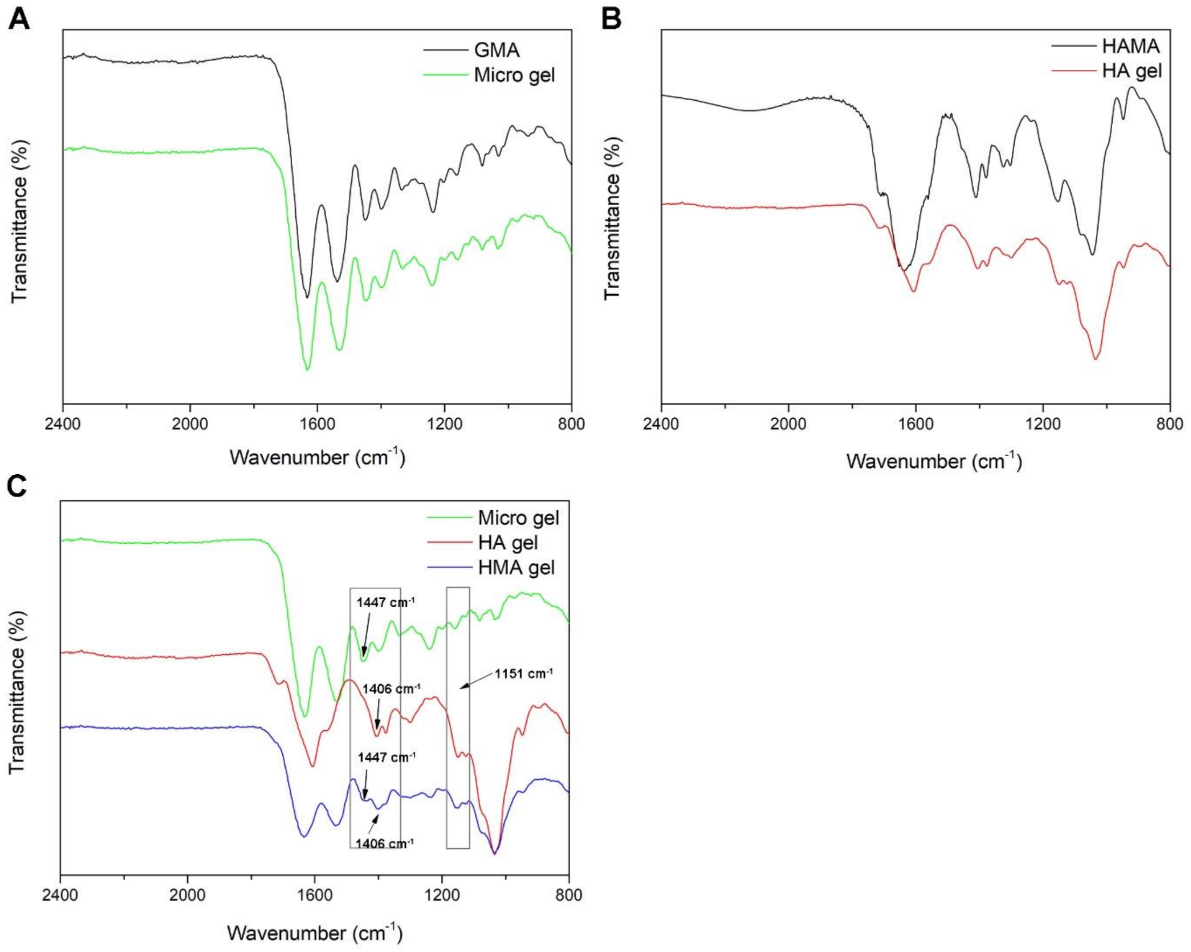

Figure S4. FTIR spectra for precursors and hydrogels. A) The compound of methacrylated gelatin (GMA) and lyophilized microspheres hydrogel. B) HA hydrogel and its precursors. C) lyophilized microspheres hydrogel, HA hydrogel and HA hydrogel containing microspheres (HMA).

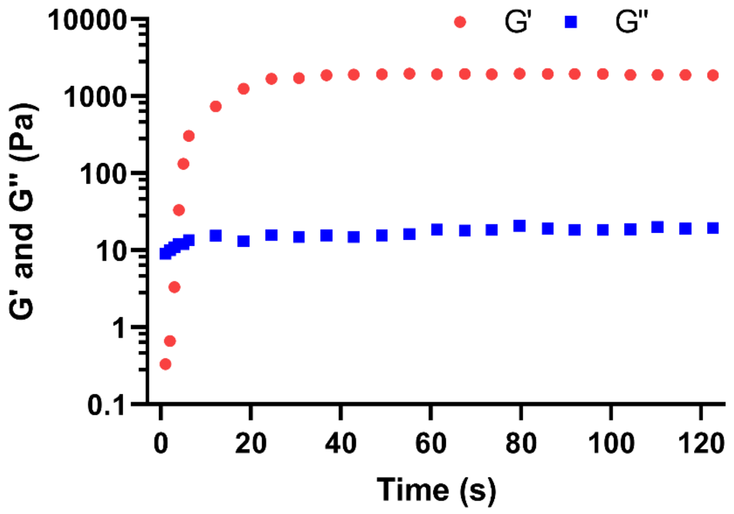

Figure S5. Rheology analysis of HA hydrogels.

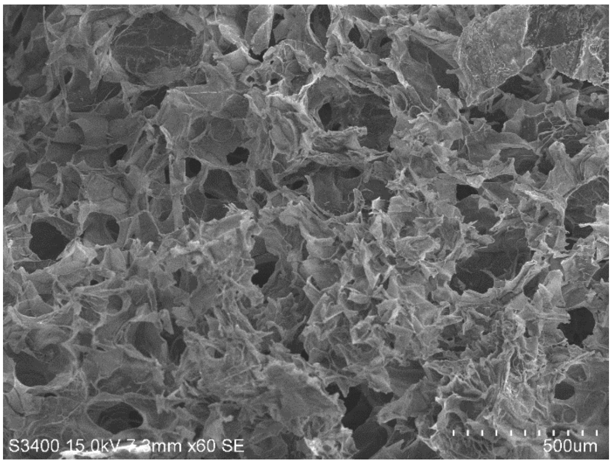

Figure S6. SEM images of lyophilized HA hydrogels.

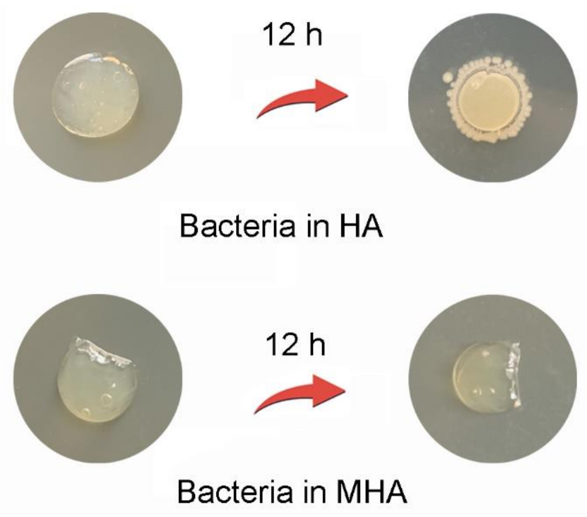

Figure S7. The culture of E. coli with (MHA) or without (HA) wrapping by microspheres in hydrogels on agar plate.

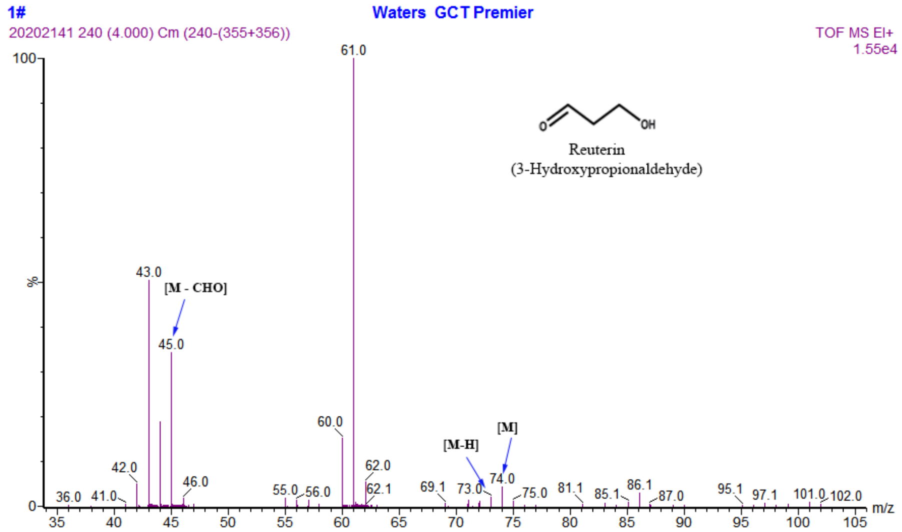

Figure S8. EI mass spectrometer of MRS broth medium after culturing with hydrogels containing Lactobacillus for overnight.

The MRS broth medium were analyzed by EI mass spectrometer after culturing with hydrogels containing Lactobacillus for overnight. As shown in Figure S, the molecular ion peak of 3-Hydroxypropionaldehyde was observed at m/z 74, which was very weak due to its extremely low concentration and instability. The peak at m/z 73 could be attributable to the ion peak of [M-H]. The signal at m/z 45 could be assigned to be a loss of aldehyde group (CHO) from the unstable reuterin. The signal present at m/z 61 was predicted to be the peak of byproduct in background solvent.

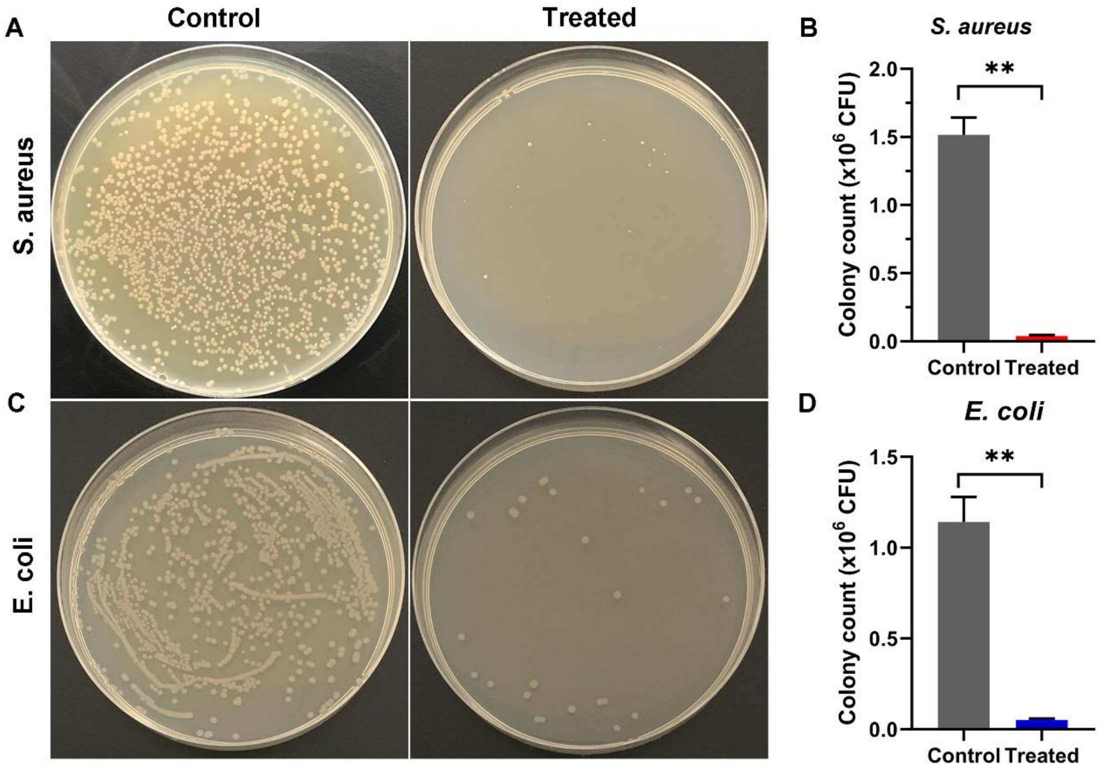

Figure S9. In vitro antibacterial activities of living hydrogels against *S. aureus* and *E. coli*. A), C) Representative Photographs of agar plates and B), D) corresponding statistical data of colonies of S. aureus and E. coli. *$p < 0.05$, **$p < 0.01$ and ***$p < 0.001$.

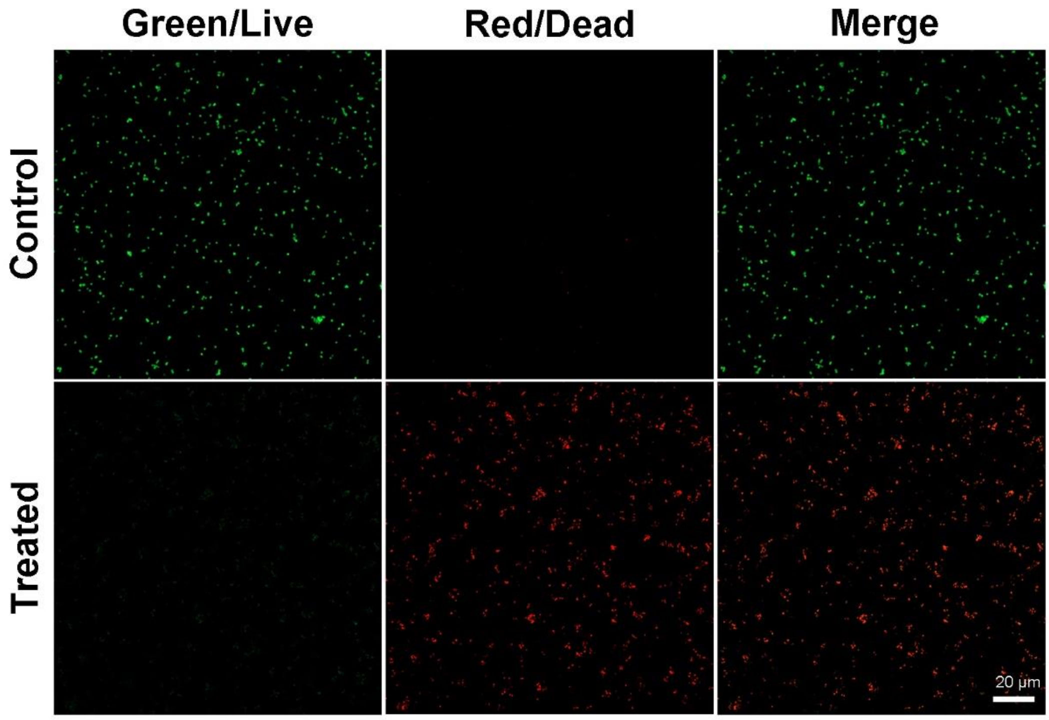

Figure S10. Fluorescent images of live/dead staining on S. aureus without or with incubating with the secretion of living *L. Reuteri* encapsulated in hydrogel. Scare bar: 20 µm.

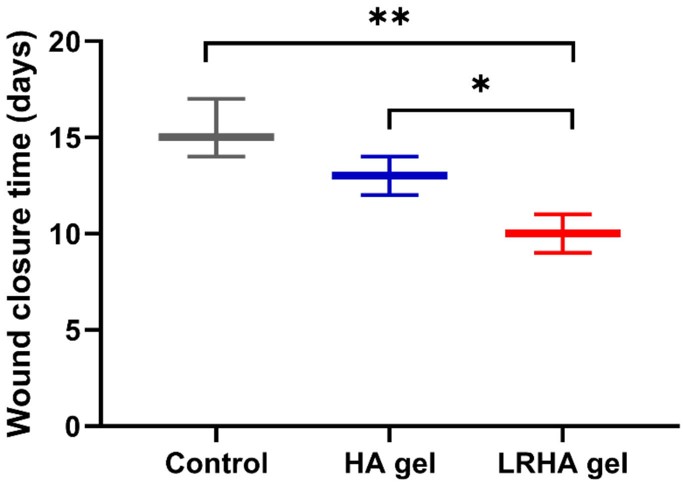

Figure S11. The wound closure time of mice treated with different methods. *p < 0.05, **p < 0.01 and ***p < 0.001.

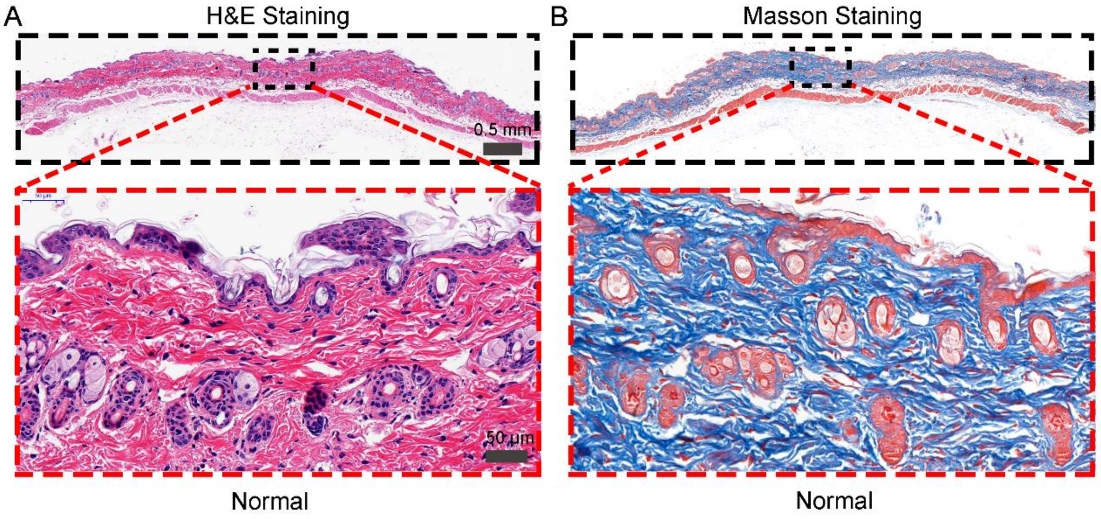

Figure S12. Micrographs of H&E stained slices and Masson stained from normal tissues.

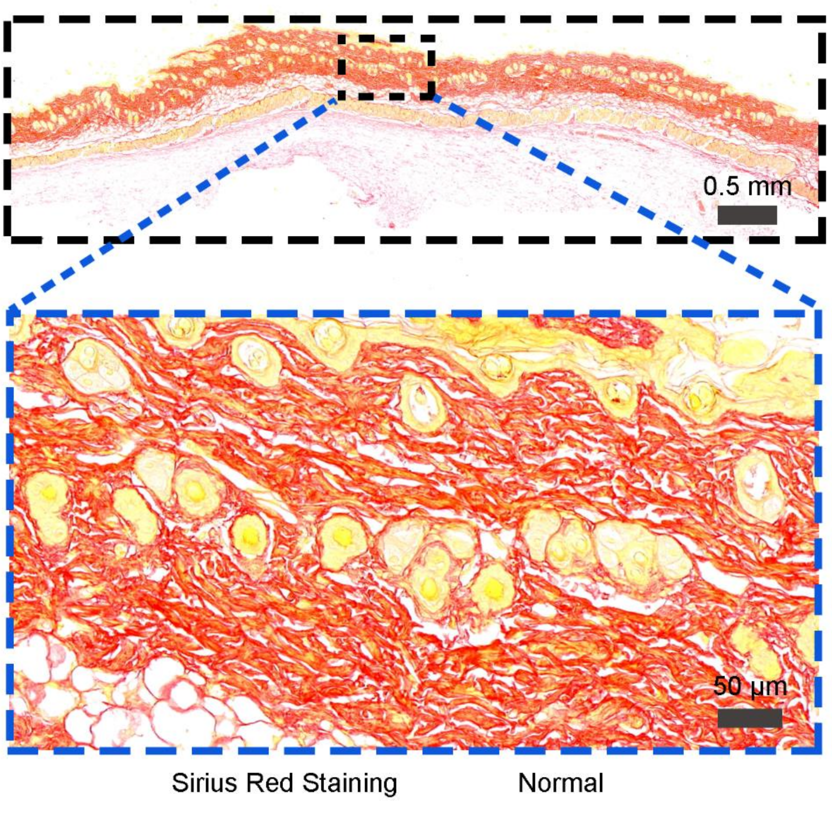

Figure S13. Micrographs of Sirius Red stained slices from normal tissues.

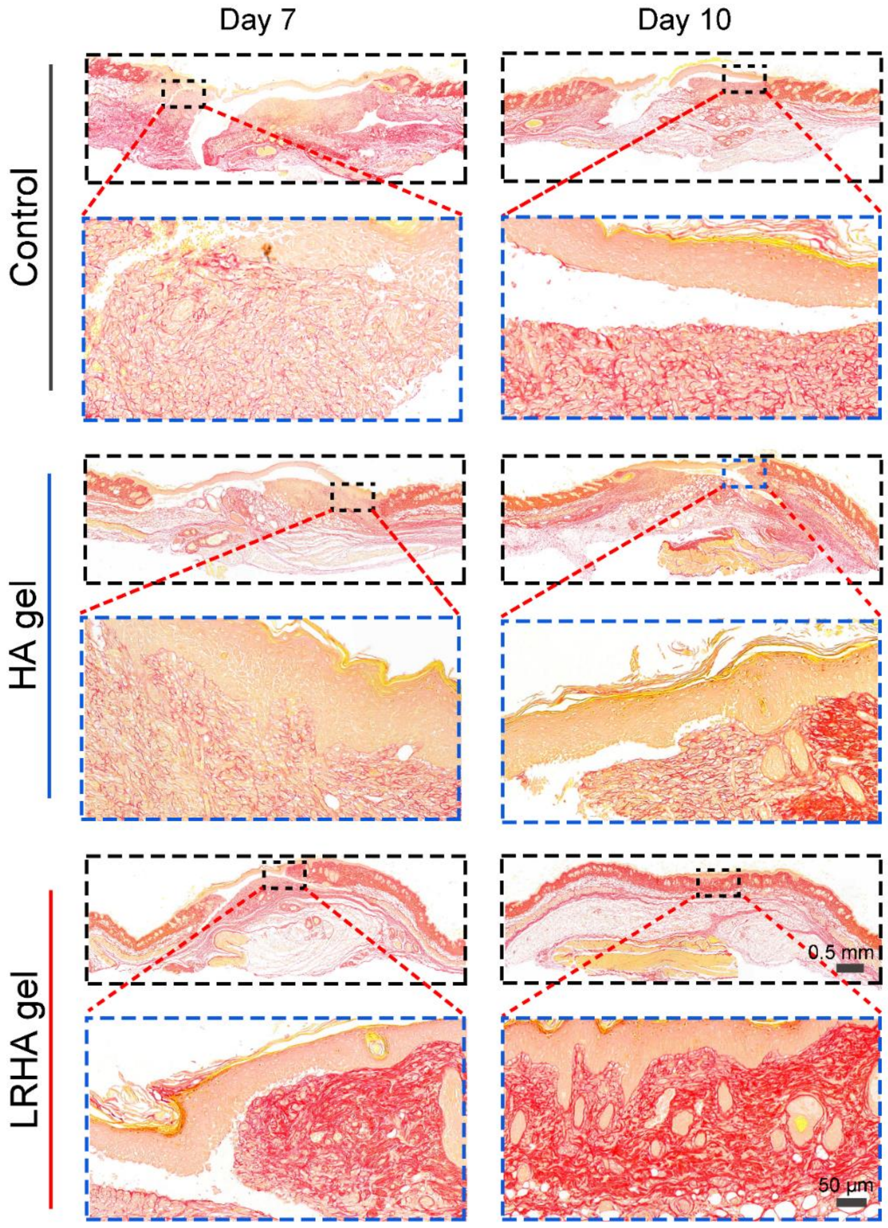

Figure S14. Micrographs of Sirius Red stained slices from control, HA and LRHA groups.

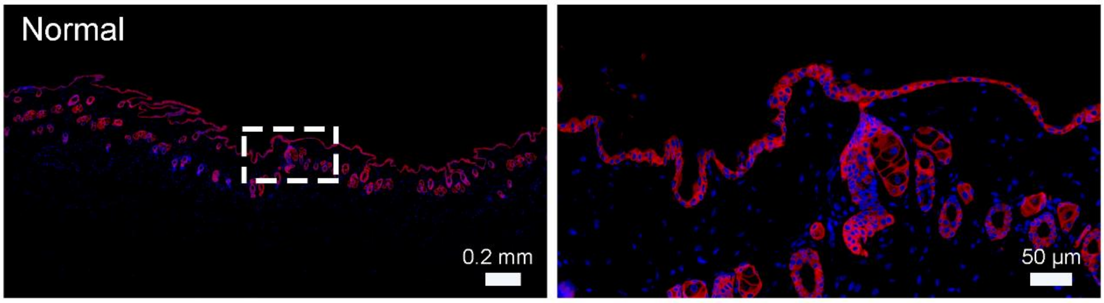

Figure S15. Representative images of immunohistochemistry staining with cytokeratin 14 for normal tissues.

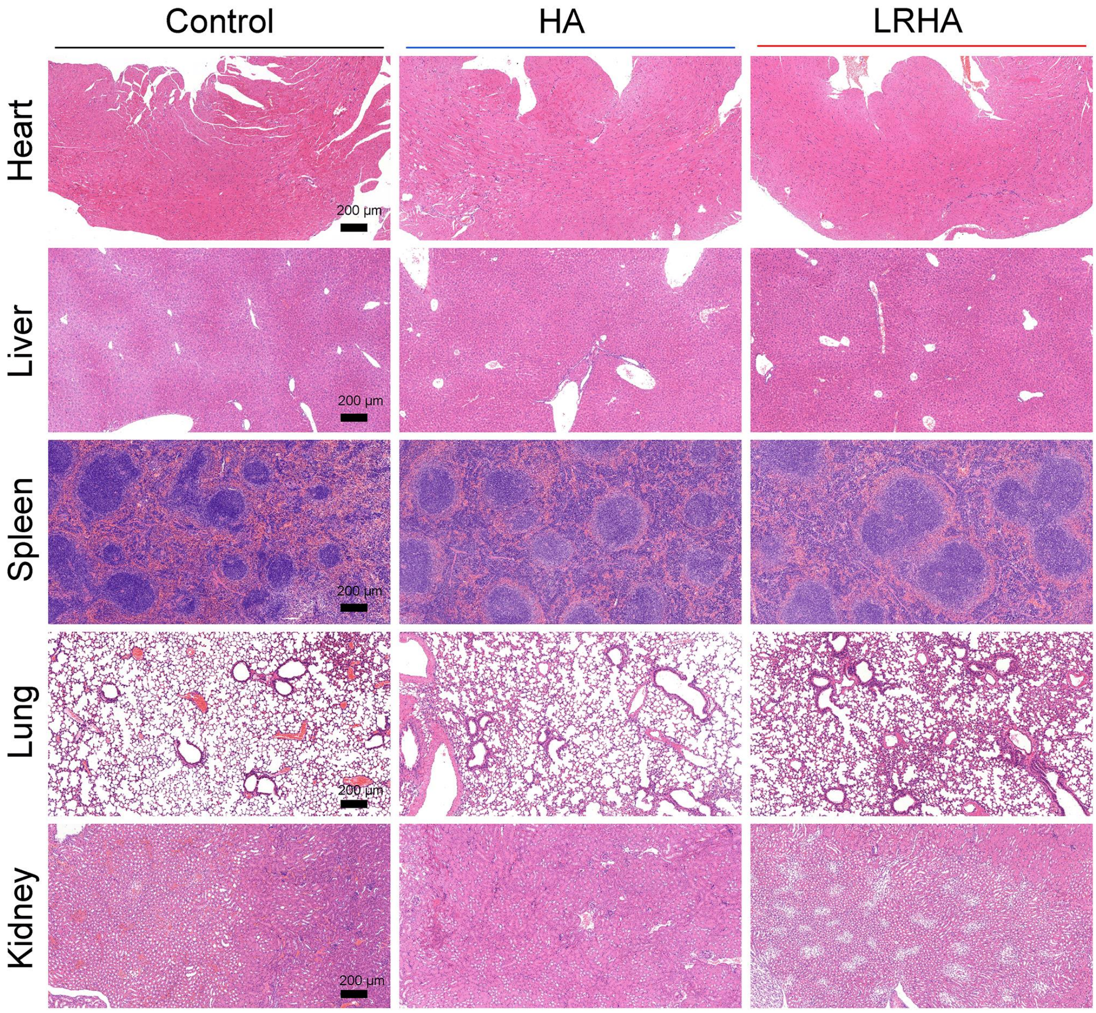

Figure S16. Micrographs of major organ tissue slices after staining with H&E in three groups.

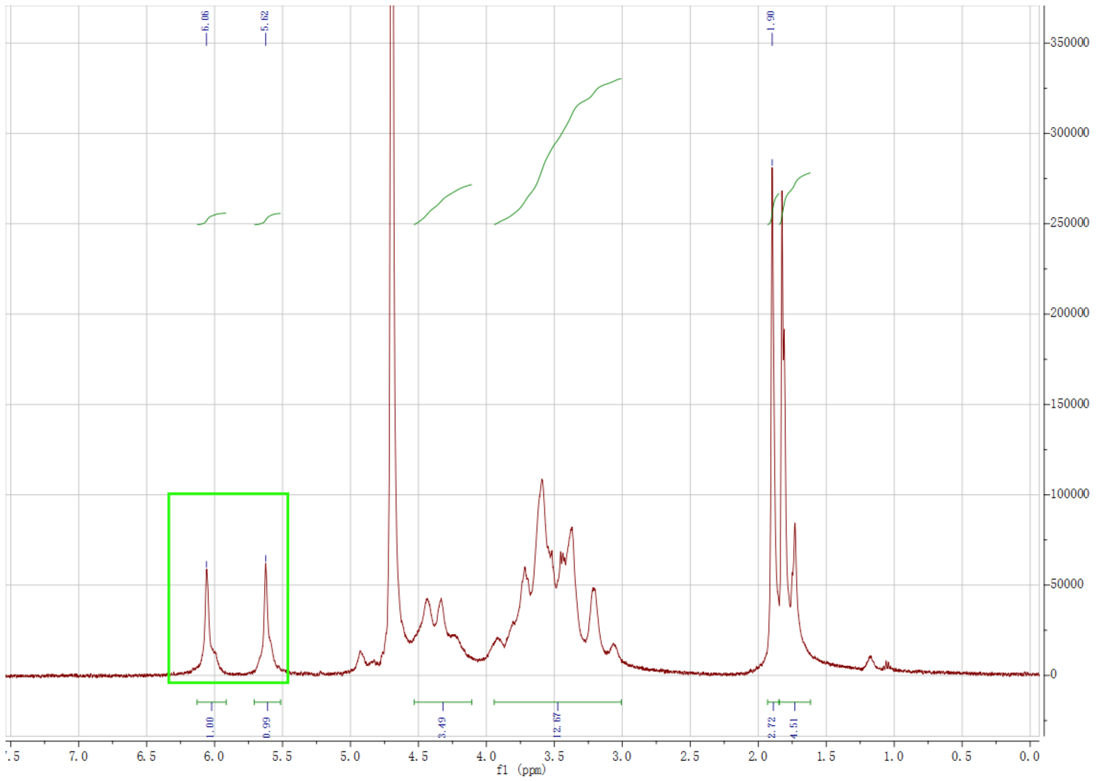

Figure S17. The ^{1}H-NMR spectra of HA-MA were recorded on a Bruker Avance 400 MHz spectrometer.

As shown in Figure S17, two chemical shifts at δ = 6.06 and δ = 5.62 that assigned to the alkene of products were observed, demonstrating successful preparation of methacrylated hyaluronic acid.
